# An Examination of the Latent Structure and Reproducibility of the Life Skills Scale for Sport in Botswana and Ghana

**DOI:** 10.3389/fpsyg.2022.858406

**Published:** 2022-04-28

**Authors:** Leapetswe Malete, Chelsi Ricketts, Sehee Kim, Tshepang Tshube, Thuso Mphela, Clement Adamba, Reginald Ocansey

**Affiliations:** ^1^Department of Kinesiology, Michigan State University, East Lansing, MI, United States; ^2^Department of Educational Administration, Michigan State University, East Lansing, MI, United States; ^3^Department of Sport Sciences, University of Botswana, Gaborone, Botswana; ^4^Department of Management, University of Botswana, Gaborone, Botswana; ^5^School of Education and Leadership, University of Ghana, Accra, Ghana; ^6^Department Physical Education and Sport, University of Ghana, Accra, Ghana

**Keywords:** positive youth development, life skills, youth sport, exploratory structural equation modeling, cultural extension

## Abstract

With the growing interest in sport-based positive youth development (PYD) programs across the African continent, there is a need to establish suitable measures to evaluate the success of these programs in fostering PYD. The Life Skills Scale for Sport (LSSS) was recently developed as a sport-specific measure of life skills development. Despite its good psychometric properties among British youth sport participants, cross-cultural evidence indicates differences in the conceptualization of the eight factors measured by the LSSS. To determine the suitability of the LSSS for use in the African youth sport context, this study examined the latent structure and reproducibility of scores produced by the scale in a sample of youth sport participants from Botswana and Ghana. Cross-sectional data from a sample of 495 youth athletes (male = 51.72%), aged 12–21 years (*M* = 16.76, *SD* = 1.58), from junior and senior secondary schools was used in this study. Confirmatory factor analysis and exploratory structural equation modeling were conducted, and conventional fit indices were used to assess model fit. Results on the original LSSS model indicated the need for model re-specification in the current sample. A re-specified LSSS, consisting of the original eight factors, but only 34 of the original 43 items, demonstrated improved fit and adequate internal consistency. Scores derived from the re-specified LSSS proved to be a valid estimate of life skills development in the current sample of youth sport participants. This has important implications for the utility of the LSSS in different cultures.

## Introduction

Converging with positive psychology, the positive youth development (PYD) framework represents a strength-based, rather than a problem-focused approach to youth development (Kelley, 2003). The advocacy of positive psychology to re-conceptualize the view of humans as “dysfunctional,” to instead being viewed as capable of optimal functioning, strength, and resiliency (Kelley, 2003), echoes the very essence of the PYD framework. At the core of the PYD approach, is an emphasis on the development of various competencies (Larson, 2000), including life skills, that are required to successfully navigate the rigors of life (World Health Organization [WHO], 1999; Larson, 2000; Danish et al., 2005). The sport context has been found to be a promising setting for PYD, given its interactive, emotional, and social nature (Danish et al., 2004; Fraser-Thomas et al., 2005; Hellison et al., 2007). These characteristics of the sport context are also considered advantageous for the social and emotional learning that is essential for one’s later success in academic, life, and career pursuits (Baciu and Baciu, 2015; Schonert-Reichl, 2017). Importantly, Forneris et al. (2015) reported that youth who participate in sport, in addition to other extracurricular activities (e.g., arts, theater), acquire more developmental assets than their non-participating counterparts. For instance, youth are considered to develop an array of life skills through sport participation, including teamwork, goal setting, interpersonal communication, and leadership (Cronin and Allen, 2017; Holt et al., 2017). However, for higher gains in outcome, life skills must be intentionally and effectively taught to foster transferability to non-sport settings (Danish et al., 2004).

Hodge et al. (2013) proposed the advancement of a comprehensive framework for determining the underlying psychological mechanisms that contribute to successful life skills development. Integrating aspects of Self-Determination Theory (SDT; Deci and Ryan, 2004) and the Life Development Intervention Model (Danish and D’Augelli, 1983), this framework posits that when the basic psychological needs of autonomy (authentic and self-directed behavior), competence (feelings of effectiveness and opportunities to express one’s capacities), and relatedness (sense of belonging), along with a needs-supportive motivational climate (where goals and behaviors related to the aforementioned basic needs are emphasized) are met, individuals experience positive psychological development and optimal psychological well-being (Hodge et al., 2013). It is within this climate that positive outcomes, including improved sport skills are developed (Papacharisis et al., 2005). Additionally, this motivational climate has been linked to further adaptive outcomes, including coach autonomy support (Cronin and Allen, 2018), psychological well-being (Steptoe and Wardle, 2017; Cronin and Allen, 2018), academic achievement (Aghajari et al., 2015), and reduced health-risk behaviors (Magnani et al., 2005). Given these reported positive outcomes, a careful and systematic assessment of life skills development through sport is imperative for facilitating sustained beneficial outcomes and an improved understanding of PYD through sport.

In light of recommendations to create more sport-specific measures of life skill development (Gould and Carson, 2008), the Life Skills Scale for Sport (LSSS; Cronin and Allen, 2017) was developed to assess the acquisition of life skill competencies through sport. The LSSS consists of 43 items and eight subscales that assess (1) Teamwork, (2) Goal setting, (3) Social skills, (4) Problem solving and decision making, (5) Emotional skills, (6) Leadership, (7) Time management, and (8) Interpersonal communication. Cronin and Allen (2017) reported the findings of four initial studies designed to evaluate the instrument’s reliability and validity, with the LSSS demonstrating good psychometric properties among samples of British youth sport participants, ages 10–21 years. These four studies, all cited in Cronin and Allen (2017), involved the development of the initial 144 items of the LSSS, with evidence provided for the items’ content validity (Study 1); the refinement of the scale to 47 items, with preliminary evidence provided for the unidimensional structure of each subscale (Study 2); further refinement of the scale to 43 items, with evidence provided for its factorial validity (Study 3) and test–retest reliability (Study 4).

Since the initial validation of the LSSS (Cronin and Allen, 2017), further evidence for the scale’s psychometric properties has been established among additional samples of British youth sport participants (Cronin and Allen, 2018; Mossman and Cronin, 2019). However, further cross-cultural examination of the LSSS has been recommended (Cronin and Allen, 2017). The scale has since been validated and adapted for use among Turkish (AÇAK and DÜZ, 2018), Korean (Lim et al., 2019), French (Sabourin et al., 2020), and Portuguese (Nascimento-Junior et al., 2020) athlete populations. Following cross-cultural adaptation, only Nascimento-Junior et al. (2020) and Sabourin et al. (2020) provided complete evidence for the structural aspects and construct validity of the LSSS. Importantly, Nascimento-Junior et al. (2020) provided support for the eight-factor model of the LSSS, along with a bi-factor model that included a total life skills factor. Within Turkish culture, only 31 of the original 43 items of the LSSS were retained, and all subscales except for problem solving and decision making satisfied model fit criteria (AÇAK and DÜZ, 2018). Of note, is that the items omitted by AÇAK and DÜZ (2018) centered around how individuals related to others (helping, motivating, and understanding others). This indicates a potential cultural difference in the conceptualization of social relationships and exchanges in Turkish culture. Cultural differences in the conceptualization of some of the items may also explain the retention of only 18 items and five factors (teamwork, goal setting, time management, social skills, and leadership) for the Korean version of the LSSS (Lim et al., 2019). While these studies highlight the usefulness of the LSSS as a cross-cultural measure of life skills development through sport, there still remains a dearth of knowledge related to the suitability of the LSSS for use among varying populations and cultures. Given the evidence of the structural differences of the LSSS when adapted cross-culturally, further research into its psychometric properties across diverse populations is crucial for the advancement of PYD and life skills research.

Interest in the role of sport in promoting PYD, as well as the use of sport-based youth development interventions, has grown tremendously over the past decade across the African continent (e.g., Sport for Development Movement; Delva et al., 2010; Langer, 2015). In fact, recent findings from a study conducted among youth sport participants in Ghana, Botswana, and Tanzania provide evidence for the utility of sport-based interventions on life skills development (Malete et al., 2022). Despite this, and other findings indicating the potential for sport participation to promote adaptive development, there remains a paucity of research examining life skills development through sport among African youth. Conducting this line of research, as well as developing and testing appropriate measurement tools, are imperative for evaluating the developmental outcomes of youth engaged in sporting activities. Importantly, assessing the psychometric properties of measurement instruments with diverse populations is a continuous process (Raykov and Marcoulides, 2011) which includes the cross-cultural validation of scales (Sousa and Rojjanasrirat, 2011). Findings on the psychometric properties of the LSSS with youth from Turkish (AÇAK and DÜZ, 2018) and Korean (Lim et al., 2019) populations, suggest that there is potential for life skills, as measured by the LSSS, to be interpreted differently across cultural contexts. Therefore, an examination of the suitability of the LSSS within the African youth sport context is warranted, prior to its full-scale use in research and program evaluation. Furthermore, the cross-cultural examination of measurement instruments presents an opportunity for data comparisons among diverse samples, thus allowing for equitable evaluation and greater generalizability within and across cultural contexts (Borsa et al., 2012).

Currently, there is only one known study that has utilized the LSSS in Botswana and Ghana (Malete et al., 2022), but the structural validity of scores produced by this tool within these two countries, and across the African continent is yet to be determined. There are strong similarities in the organization and structure of youth sport in Botswana and Ghana, but potential cultural differences in the value placed on sport. Youth sports in the two countries are mostly offered by schools and there are limited out of school or community-based programs. Ghana, however, has a larger and seemingly more developed amateur and youth sport platform compared to Botswana. For instance, Ghana has a significant number of youth soccer players who compete in professional soccer leagues in Europe and other regions of the world. These similarities and differences make the two countries interesting comparative cases for PYD research and testing of the structural validity of the LSSS.

### The Present Study

The purpose of this study was to examine the latent structure and reproducibility of scores produced by the LSSS among youth sport participants in two African countries (Botswana and Ghana). The two countries were deemed ideal for this research because they are located in separate regions of Africa that are considered to be culturally diverse. Botswana is located in southern Africa while Ghana is located in West Africa. Ascertaining whether the LSSS has good psychometric properties in these two regions of Africa is an important step in determining the tool’s appropriateness for use when conducting sport-based PYD research across contexts. Current evidence on the psychometric properties of the LSSS shows that this tool has promise when used in other cultures. Testing existing tools for their appropriateness and relevance to different contexts is key to monitoring and evaluation of youth sport programs and their effectiveness in fostering PYD.

## Materials and Methods

### Participants

This cross-sectional study targeted a purposive sample of 718 youth athletes, aged 12–21 years (*M* = 16.76, *SD* = 1.58), from public, middle, and high (junior and senior secondary) schools in Botswana and Ghana taking part in a sport-based PYD research project. A total of 495 participants were retained for examining the latent structure and reproducibility of the LSSS. A total of 223 of the original 718 participants were excluded from this study (Botswana = 120; Ghana = 103) because of incomplete demographic information and unverifiable data about their athlete status. Approximately 52% of the participants in the retained data were male and all participants were of African descent. Botswana had 342 participants (183 males and 159 females), aged 12–20 years (*M* = 16.81, *SD* = 1.56), from seven public schools in Gaborone, the capital. Ghana had 153 participants (73 males and 80 females) aged 12–21 years (*M* = 16.64, *SD* = 1.62) from four public schools in Accra, the capital. Participants played a wide range of sports, with the top three sports being soccer (∼30%), volleyball (∼17%), and track field (∼11%). Other sports were handball, basketball, cricket, karate, boxing, softball, badminton, tennis, field hockey, and netball.

### Procedures

Permission to conduct this study was obtained from the Institutional Review Boards (IRB) of partner universities and the government ministries (departments) responsible for youth, sport, and education in each country. Additional approvals were obtained from the schools. Consent and assent were received from all parents/guardians and participants prior to enrollment in the study. Parental consent and student assent forms were distributed to participants who identified as school athletes at their schools. Participants took these forms home along with a letter of introduction that stated the objectives of the study. The signed consent and assent forms were returned to the project team prior to survey administration. All selected participants agreed to participate and also received parental consent to complete the surveys. Surveys were completed in school halls or classrooms with the help of trained undergraduate research assistants. Participants completed questionnaires themselves under the guidance of research assistants and returned questionnaires upon completion. Test administration lasted approximately 30 min.

### Measures

#### Demographic Information

A demographic information questionnaire was used to collect data on age, gender, and type of sport played.

#### Life Skills Development

Life skills development through sport was assessed using the Life Skills Scale for Sport (LSSS; Cronin and Allen, 2017). The 43 items of the LSSS were developed to measure eight subscales: Teamwork (7 items), Goal-setting (7 items), Social skills (5 items), Problem solving and decision making (4 items), Emotional skills (4 items), Leadership (8 items), Time management (4 items), and Interpersonal communication (4 items). These items are anchored on a five-point Likert-type scale ranging from 1 (not at all) to 5 (very much). Participants were asked to rate how much they think playing sport had helped them to develop or learn the various life skills represented by the different items. Cronbach’s alpha for each of the eight subscales from the initial study of the instrument’s development and validation were above 0.70 (Cronin and Allen, 2017). Internal consistency for the eight-factor structure of the LSSS in the current sample ranged between 0.69 and 0.83.

### Data Analyses

#### Factorial Validity

Confirmatory factor analysis (CFA) and exploratory structural equation modeling (ESEM) were conducted to test the hypothesized factor structure of the LSSS in Botswana and Ghana. While CFA provides a useful tool to test theoretically established constructs, there are concerns regarding its restrictiveness. CFA permits items to load only on their intended factor, with potential cross-loadings with other factors being restricted to zero (Marsh et al., 2014; Tomás et al., 2014). This requirement is likely to be restrictive for multidimensional measures in social and psychological contexts, as it is likely for items to load on the other factors at least to some degree (Morin et al., 2016). As such, a CFA approach that restricts cross-loadings to zero is argued to not account for the multidimensional nature of instruments, having limitations including poor model fit, as well as high correlations between factors that threaten the discriminant validity (McCrae et al., 1996; Marsh et al., 2009, 2010; Schmitt and Sass, 2011). As an alternative, ESEM provides a more flexible analytical tool to define the factor structure. In ESEM, the restrictions imposed in CFA are relaxed and the possible cross-loadings of items are allowed to be freely estimated (Asparouhov and Muthén, 2009; Marsh et al., 2009, 2010, 2014), while still allowing advanced statistical techniques that are enabled in CFA to be applied (e.g., estimation of factor loadings’ standard errors; Asparouhov and Muthén, 2009; Marsh et al., 2009). The ESEM was estimated with oblique geomin rotation. Analytical procedures were conducted using the R software packages “psych (Revelle, 2018)” and “lavaan (Rosseel, 2012).”

#### Analytic Estimator

The robust maximum-likelihood (MLR) estimator was applied to all analyses to deal with possible non-normality in the measures. The missing data were handled using the full-information maximum-likelihood (FIML) estimator (Enders and Bandalos, 2001).

#### Goodness-of-Fit

The models were evaluated using the following model fit statistics: chi-square goodness-of-fit tests, the comparative fit index (CFI), the Tucker–Lewis index (TLI), the root-mean-square error of approximation (RMSEA), and the standardized root-mean-square residual (SRMR). CFI and TLI are considered to be acceptable when values are greater than 0.90 (Hu and Bentler, 1999; Kline, 2011); RMSEA values less than 0.06 are considered to be acceptable (Hu and Bentler, 1999); SRMR values equal to or less than 0.08 indicate reasonable fit (Hu and Bentler, 1999). Additionally, models were compared using the Akaike Information Criterion (AIC) and the Bayesian Information Criterion (BIC), in which a smaller value indicates a better fitting model (Akaike, 1987; Raftery, 1995).

#### Internal Consistency

The internal consistency of the eight LSSS subscales was assessed with Cronbach’s alpha. A Cronbach’s alpha greater than 0.70 is considered to be good and an alpha between 0.60 and 0.70 is considered acceptable (Kline, 2000).

## Results

### Descriptive Analyses

Table 1 presents the specific items designed to measure each LSSS subscale as well as the descriptive statistics of each item. Each item had a low proportion of missing values (ranging from 0.002 to 0.055%; 0.019% on average). The mean of each item ranged between 3.63 (item 15) and 4.54 (item 4). Skewness and kurtosis values of all items were within the acceptable range for normality assumption between −2 and +2 for skewness and between −7 and +7 for kurtosis (Bryne, 2010; Hair et al., 2010).

**TABLE 1:** Descriptive statistics for the life skill scale for sport items.

Factors and Items	Item No.	*M*	*SD*	Skewness	Kurtosis	Missing%
**Teamwork**						
Work well within a team/group.	1	4.42	0.88	–1.72	2.78	0.002
Help another team/group member perform a task.	2	4.00	1.07	–0.99	0.30	0.008
Accept suggestions for improvement from others.	3	4.26	0.95	–1.23	0.91	0.012
Work with others for the good of the team/group.	4	4.54	0.76	–1.97	4.15	0.012
Help build team/group spirit.	5	4.50	0.81	–1.87	3.82	0.022
Suggest to team/group members how they can improve their performance.	6	3.93	1.03	–0.72	–0.15	0.006
Change the way I perform for the benefit of the team/group.	7	4.14	1.04	–1.15	0.58	0.010
**Goal setting**						
Set goals so that I can stay focused on improving.	8	4.42	0.82	–1.58	2.62	0.006
Set challenging goals.	9	4.07	0.99	–0.97	0.42	0.012
Check progress toward my goals.	10	4.05	0.99	–0.87	0.02	0.038
Set short-term goals in order to achieve long-term goals.	11	3.81	1.16	–0.78	–0.28	0.008
Remain committed to my goals.	12	4.22	0.99	–1.38	1.40	0.010
Set goals for practice.	13	4.09	0.99	–0.95	0.25	0.010
Set specific goals.	14	4.07	1.03	–0.90	–0.06	0.014
**Social skills**						
Start conversation.	15	3.63	1.25	–0.54	–0.81	0.010
Interact in various social settings.	16	3.84	1.14	–0.72	–0.39	0.006
Help others without them asking for help.	17	3.86	1.14	–0.78	–0.18	0.010
Get involved in group activities.	18	4.20	1.01	–1.24	0.88	0.012
Maintain close friendships.	19	4.27	0.97	–1.32	1.19	0.010
**Problem solving and decision making**						
Think carefully about a problem.	20	4.10	0.95	–1.06	0.71	0.012
Compare each possible solution in order to find the best one.	21	3.96	1.03	–0.78	–0.14	0.008
Create as many possible solutions to a problem as possible.	22	3.83	1.05	–0.71	–0.01	0.018
Evaluate a solution to a problem.	23	3.97	1.03	–0.89	0.33	0.055
**Emotional skills**						
Know how to deal with my emotions.	24	4.07	1.12	–1.20	0.63	0.016
Use my emotions to stay focused.	25	3.95	1.15	–1.02	0.22	0.026
Understand that I behave differently when emotional.	26	3.95	1.16	–1.00	0.14	0.040
Notice how I feel.	27	4.07	1.09	–1.18	0.70	0.024
**Leadership**						
Know how to positively influence a group of individuals.	28	4.00	1.11	–0.96	0.11	0.026
Organize team/group members to work together.	29	4.20	1.02	–1.27	1.04	0.022
Know how to motivate others.	30	4.25	0.93	–1.22	0.93	0.034
Help others solve their performance problems.	31	3.94	1.04	–0.79	–0.04	0.028
Consider the individual opinions of each team/group member.	32	3.94	1.04	–0.80	–0.06	0.030
Be a good role model for others.	33	4.33	0.88	–1.35	1.42	0.016
Set high standards for the team/group.	34	4.22	0.95	–1.13	0.69	0.030
Recognize other people’s achievements.	35	4.22	0.95	–1.20	0.93	0.022
**Time management**						
Manage my time well.	36	4.31	0.92	–1.34	1.37	0.018
Assess how much time I spend on various activities.	37	4.08	0.97	–0.92	0.27	0.024
Control how I use my time.	38	4.17	0.96	–1.06	0.54	0.026
Set goals so that I use my time effectively.	39	4.21	1.00	–1.22	0.79	0.030
**Interpersonal communication**						
Speak clearly to others.	40	4.20	0.99	–1.30	1.28	0.018
Pay attention to what someone is saying.	41	4.39	0.91	–1.71	2.84	0.024
Pay attention to people’s body language.	42	4.05	1.11	–1.11	0.49	0.026
Communicate well with others.	43	4.48	0.88	–1.95	3.61	0.024

*Missing% = the proportion of missing values for each item.*

### Confirmatory Factor Analysis and Exploratory Structural Equation Modeling Fit of the Life Skills Scale for Sport Model

The summary of the model fit indices for the eight-factor LSSS model with 43 items is presented in the upper section of Table 2. The eight-factor CFA model presented χ^2^(832) = 1344.76 (*p* < 0.001); CFI = 0.865; TLI = 0.853; RMSEA = 0.045; and SRMR = 0.050. Although the RMSEA and the SRMR met the cut-off criteria for the goodness of fit, the CFI and TLI values were below the threshold of 0.90. The ESEM model presented a better fit: χ^2^(587) = 960.11 (*p* < 0.001), CFI = 0.924, TLI = 0.883, RMSEA = 0.040, and SRMR = 0.029. Although all the goodness-of-fit indices considered had improved, the TLI still remained slightly below the acceptable threshold of 0.90.

**TABLE 2:** Indices of model fit for the life skills scale for sport.

Model	χ^2^	*df*	*p*	CFI	TLI	RMSEA	SRMR	AIC	BIC
CFA	1344.76	832	<0.001	0.865	0.853	0.045	0.050	54123.21	54783.32
ESEM	960.11	587	<0.001	0.924	0.883	0.040	0.029	53998.48	55688.72
CFA (re-specified)	712.42	499	<0.001	0.917	0.907	0.040	0.045	42502.53	43049.13
ESEM (re-specified)	435.67	317	<0.001	0.962	0.933	0.034	0.023	42466.29	43778.12

*N = 495. CFI, comparative fit index; TLI, Tucker–Lewis index; RMSEA, root-mean-square error of approximation; SRMR, standardized root-mean-square residual; AIC, Akaike information criterion; BIC, Bayesian information criterion.*

The correlations among factors also remarkably decreased in the ESEM model (see Table 3). Inter-factor correlations of the CFA model ranged from 0.25 (Teamwork – Time management) to 0.69 (Leadership – Interpersonal communication). Particularly, except for correlations between Teamwork and Time management (0.25), and between Social skills and Time management (0.39), all correlations were close to 0.5 or greater. In contrast, inter-factor correlations in the ESEM model were far smaller, ranging from 0.084 (Teamwork – Time management) to 0.46 (Goal setting – Problem solving and decision making).

**TABLE 3:** Inter-factor correlations for the confirmatory factor analysis (CFA) and exploratory structural equation modeling (ESEM) models.

	1	2	3	4	5	6	7	8
**CFA Model**								
1. TW	−							
2. GS	0.545***	−						
3. SS	0.544***	0.470***	−					
4. PS	0.461***	0.609***	0.518***	−				
5. ES	0.481***	0.543***	0.572***	0.499***	−			
6. LS	0.624***	0.594***	0.592***	0.627***	0.578***	−		
7. TM	0.247**	0.543***	0.387***	0.539***	0.497***	0.553***	−	
8. IC	0.463***	0.466***	0.555***	0.527***	0.602***	0.689***	0.584***	−
**ESEM Model**								
1. TW	−							
2. GS	0.246*	−						
3. SS	0.271**	0.249*	−					
4. PS	0.233*	0.455**	0.320**	−				
5. ES	0.195	0.305**	0.296*	0.273*	−			
6. LS	0.242*	0.354**	0.250**	0.374**	0.246*	−		
7. TM	0.080	0.384***	0.260***	0.422***	0.255**	0.297***	−	
8. IC	0.264	0.209	0.326**	0.358**	0.250	0.288**	0.428**	−
α	0.69	0.79	0.74	0.74	0.71	0.80	0.83	0.72

TW, teamwork; GS, goal setting; SS, social skills; PS, problem solving and decision making; ES, emotional skills; LS, leadership skills; TM, time management; IC, interpersonal communication. *p < 0.05, **p < 0.01, ***p < 0.001.

Although the initial eight-factor model’s overall fit statistics were generally considered adequate, Brown (2006) noted that judging acceptability of the model should not rely solely on global fit indices, recommending that researchers further examine the relationships among the item components and their target factors to detect local misfit. Table 4 presents the standardized factor loadings of the items in the CFA and ESEM models. The factor loadings of the CFA were generally moderate to high, ranging between 0.411 (item 2 for Teamwork) and 0.830 (item 38 for Time management), except for two items. Specifically, items 3 (My sport has taught me to accept suggestions for improvement from others) and 7 (My sport has taught me to change the way I perform for the benefit of the team/group), that were designed to measure Teamwork, showed relatively low loadings below 0.4 (0.379 and 0.382, respectively). The corresponding ESEM solution rejects the hypothesized factor structure with our data from Botswana and Ghana. Of the 43 items, seven items presented low loadings below 0.3 on the factor the item were intended to measure (items 2, 3, 6, and 7 for Teamwork; item 18 for Social skills; item 29 and 35 for Leadership); four items had their primary loadings on factors other than their target factor (items 2 and 6 for Teamwork; item 29 and 35 for Leadership); eight items had relatively large cross-loadings greater than 0.2 (items 2, 6, and 7 for Teamwork: item 18 for Social skills; items 28, 29, 33, and 35 for Leadership). This result suggests that there are improper measures of the LSSS subscales and substantial overlap between the subscales, challenging the suitability of the LSSS structure in Botswana and Ghana youth sport contexts.

**TABLE 4:** Standardized factor loadings for the life skills scale for sport.

Factors and Items	CFA	ESEM							
		
		TW	GS	SS	PS	ES	LS	TM	IC
TW1	0.658***	0.671***	0.023	0.057	0.057	–0.023	–0.057	0.011	0.103
TW2	0.411***	0.213**	0.111	0.049	–0.058	0.062	0.242**	0.018	–0.036
TW3	0.379***	0.227**	0.132	–0.080	0.163	0.100	0.061	0.011	–0.060
TW4	0.592***	0.648***	0.017	–0.009	0.094	–0.021	0.045	–0.037	–0.059
TW5	0.610***	0.566***	0.116	0.134	–0.077	0.030	–0.055	0.020	0.045
TW6	0.477***	0.204*	0.023	0.061	0.056	0.184	0.275***	–0.013	–0.057
TW7	0.382***	0.255**	0.128	–0.141	–0.047	0.198	0.229**	–0.019	–0.090
GS8	0.527***	0.130	0.427**	–0.036	0.062	0.135	–0.033	–0.017	0.023
GS9	0.566***	–0.029	0.563***	–0.055	0.008	0.007	0.142	0.003	–0.042
GS10	0.637***	–0.044	0.578***	0.052	0.012	0.088	0.112	0.008	–0.052
GS11	0.536***	–0.038	0.438***	0.037	0.014	0.154	–0.009	0.050	0.050
GS12	0.629***	0.030	0.593***	–0.010	0.121	–0.101	–0.030	0.020	0.101
GS13	0.625***	0.148	0.497***	0.114	–0.017	–0.007	–0.068	0.120	0.062
GS14	0.599***	0.115	0.497***	0.023	0.071	–0.020	–0.012	0.097	–0.054
SS15	0.648***	0.058	0.011	0.637***	0.087	0.073	–0.003	–0.056	–0.020
SS16	0.687***	0.009	0.000	0.781***	–0.009	0.000	–0.026	0.026	0.018
SS17	0.615***	–0.053	0.061	0.423***	0.157	0.053	0.157*	–0.014	0.009
SS18	0.552***	0.277**	–0.006	0.290**	0.011	–0.038	0.084	0.106	0.103
SS19	0.494***	0.056	–0.041	0.373***	–0.064	0.132	0.121	–0.007	0.071
PS20	0.578***	0.058	0.058	0.079	0.504	–0.007	–0.027	–0.011	0.038
PS21	0.669***	0.109	0.028	0.022	0.604*	0.075	0.008	–0.007	–0.046
PS22	0.744***	–0.019	–0.010	0.010	0.714***	0.029	0.038	0.019	0.029
PS23	0.608***	–0.033	0.036	0.039	0.596***	–0.070	–0.020	0.105	–0.034
ES24	0.645***	0.069	0.026	0.014	0.006	0.385**	0.055	0.171	0.152
ES25	0.653***	0.064	0.093	0.059	0.016	0.556***	–0.106	0.113	0.059
ES26	0.578***	–0.078	0.009	0.043	0.026	0.651***	0.009	0.000	0.009
ES27	0.574***	0.020	–0.035	0.140	0.105	0.430***	0.064	0.017	0.064
LS28	0.646***	0.097	–0.107	0.028	0.115	0.042	0.356**	0.105	0.247
LS29	0.663***	0.271*	–0.040	0.093	0.029	–0.081	0.281*	0.137	0.288
LS30	0.586***	–0.055	0.118	0.109	0.047	–0.088	0.463***	0.002	0.192
LS31	0.616***	–0.048	0.077	0.038	0.038	0.000	0.722***	0.058	–0.048
LS32	0.573***	0.187	0.047	–0.047	0.099	0.030	0.464***	0.037	0.016
LS33	0.472***	–0.029	0.079	–0.063	–0.031	0.095	0.324**	0.016	0.254
LS34	0.591***	0.165	–0.035	–0.033	0.165	0.158	0.348**	–0.017	0.103
LS35	0.432***	–0.023	0.000	–0.008	0.117	0.240*	0.204*	0.033	0.110
TM36	0.688***	0.041	0.014	−0.118*	0.076	0.065	–0.086	0.627***	0.144
TM37	0.719***	0.052	–0.026	0.047	–0.037	0.103	0.038	0.754***	–0.095
TM38	0.830***	–0.052	0.021	0.052	0.031	–0.052	0.010	0.796***	0.042
TM39	0.726***	–0.070	0.147*	–0.020	0.123	–0.057	0.071	0.581***	0.028
IC40	0.742***	–0.059	0.054	0.083	0.037	0.041	0.031	0.139	0.590**
IC41	0.637***	0.011	0.033	–0.011	0.055	0.033	–0.055	–0.044	0.675***
IC42	0.531***	0.021	0.139	0.014	0.136	0.167*	–0.012	–0.032	0.336*
IC43	0.633***	0.118	–0.012	0.049	–0.041	0.047	0.073	0.030	0.557***

TW, teamwork; GS, goal setting; SS, social skills; PS, problem solving and decision making; ES, emotional skills; LS, leadership skills; TM, time management; IC, interpersonal communication. *p < 0.05, **p < 0.01, ***p < 0.001.

We therefore re-specified the model based on the magnitude of factor loadings and theoretical considerations. The items that had standardized factor loadings on their target factor less than 0.30 and/or had cross-loadings less than 0.15 difference from the primary factor loading were selected (Worthington and Whittaker, 2006). Nine items (items 2, 3, 6, and 7 for Teamwork; item 18 for Social skills; items 28, 29, 33, and 35 for Leadership) fell into the criteria. As eight out of the nine items that were identified as ‘problematic’ were originally designed to measure Teamwork or Leadership, we conducted additional explorations of the two subscales before making the final decision. Table 4 above shows that of the seven items (i.e., items 1–7) intended to measure Teamwork, only three (items 1, 4, and 5) had adequate loadings in the ESEM solution. As such, we suspected that the subscale of Teamwork might consist of two subfactors in the context of this study. If that were the case, it would be better to split Teamwork into two adequate subfactors rather than to simply remove the items with small loadings. This hypothesis was tested using Exploratory Factor Analysis (EFA; principal component analysis with varimax rotation). We performed EFA on the seven items for Teamwork but there was no statistical evidence of the presence of subfactors. Furthermore, of the four items for Teamwork that had loadings smaller than 0.30 (i.e., items 2, 3, 6, and 7), three (i.e., items 2, 6, and 7) had noticeable cross-loadings on Leadership. We hence conducted EFA again on the 15 items (seven items for Teamwork and eight items for Leadership) to see if there was any hidden factor for the current data. The EFA generated two categories; one factor was defined by items 1–7 and the other factor was defined by the remaining eight items (i.e., items 28–35). That is, there was no evidence of a hidden factor. We therefore concluded that the selected nine items did not work as expected in the hypothesized eight-factor model in the context of this study. Rather than defining previously unrevealed factors, the LSSS structure was respecified without the nine items.

### Fit of the Re-specified Life Skills Scale for Sport Model

The re-specified CFA solution provided an improved level of fit compared to the original hypothesized model and all indices met the fit indices criteria [see Table 2: χ^2^(499) = 712.42 (*p* < 0.001); CFI = 0.917; TLI = 0.907; RMSEA = 0.040; and SRMR = 0.045]. Also, AIC and BIC indices indicated that the re-specified model was preferable compared to the original model. The factor loadings of the revised CFA model presented in Table 5 revealed that all items adequately defined their target factors. The factor loadings of the CFA solution ranged from 0.524 (item 8 for Goal setting) to 0.829 (item 38 for Time management). As expected, the ESEM resulted in better and smaller inter-factor correlations than the corresponding CFA model [see Table 6; χ^2^(317) = 435.67 (*p* < 0.001); CFI = 0.962; TLI = 0.933; RMSEA = 0.034; and SRMR = 0.023]. The standardized factor loadings for the revised ESEM model show that the items have salient loadings on their target factors without noticeable cross-loadings, despite three items (item 19 for Social skills; item 34 for Leadership; item 42 for Interpersonal communication) having relatively low loadings below 0.4 (see Table 5).

**TABLE 5:** Standardized factor loadings for the re-specified model of the life skills scale for sport.

Factors and Items	CFA	ESEM							
		
		TW	GS	SS	PS	ES	LS	TM	IC
TW1	0.726***	0.683***	0.000	0.046	0.046	–0.034	–0.023	0.023	0.103
TW4	0.632***	0.643***	0.003	–0.026	0.100	–0.013	0.060	–0.045	–0.049
TW5	0.663***	0.579***	0.126	0.115	–0.101	0.028	–0.032	0.022	0.030
GS8	0.524***	0.132	0.438***	–0.041	0.056	0.142	–0.040	–0.022	–0.012
GS9	0.573***	–0.005	0.588***	–0.059	–0.009	0.017	0.115	–0.015	–0.057
GS10	0.641***	–0.050	0.628***	0.050	–0.010	0.091	0.070	–0.020	–0.050
GS11	0.533***	–0.061	0.476***	0.046	0.001	0.117	–0.047	0.033	0.075
GS12	0.630***	–0.005	0.619***	–0.019	0.072	–0.116	–0.016	0.000	0.137
GS13	0.620***	0.117	0.500***	0.114	–0.038	–0.023	–0.044	0.100	0.083
GS14	0.599***	0.088	0.532***	0.012	0.060	0.013	–0.032	0.068	–0.063
SS15	0.684***	0.060	0.008	0.619***	0.086	0.061	0.030	–0.050	–0.017
SS16	0.734***	0.009	0.000	0.806***	–0.009	–0.009	–0.026	0.035	0.009
SS17	0.594***	–0.062	0.055	0.407***	0.187	0.047	0.137	–0.025	0.038
SS19	0.466***	0.052	–0.017	0.357***	–0.046	0.139	0.093	–0.014	0.059
PS20	0.574***	0.012	0.051	0.073	0.558*	–0.002	–0.062	–0.016	0.046
PS21	0.666***	0.093	0.009	0.011	0.652**	0.079	–0.006	–0.004	–0.049
PS22	0.748***	–0.010	–0.010	0.019	0.668***	–0.01	0.048	0.048	0.057
PS23	0.611***	–0.024	0.039	0.042	0.544***	–0.078	0.004	0.128	–0.029
ES24	0.641***	0.105	–0.020	–0.006	0.030	0.429***	0.079	0.178*	0.101
ES25	0.657***	0.098	0.095	0.050	–0.011	0.530***	–0.068	0.116	0.043
ES26	0.580***	–0.078	0.017	0.017	0.026	0.686***	0.017	–0.026	0.009
ES27	0.572***	0.016	–0.034	0.138	0.111	0.429***	0.053	0.012	0.067
LS30	0.611***	–0.035	0.104	0.099	0.062	–0.055	0.416***	–0.006	0.195
LS31	0.708***	0.000	0.01	0.000	–0.01	0.000	0.911***	0.019	–0.01
LS32	0.607***	0.195*	0.062	–0.055	0.089	0.025	0.412***	0.013	0.073
LS34	0.547***	0.210*	–0.007	–0.029	0.185	0.143	0.253**	–0.001	0.075
TM36	0.688***	0.047	0.019	–0.105	0.074	0.052	–0.099	0.637***	0.128
TM37	0.721***	0.051	–0.023	0.046	–0.023	0.097	0.040	0.735***	–0.084
TM38	0.829***	–0.031	0.000	0.063	0.021	–0.052	0.031	0.809***	0.031
TM39	0.726***	–0.067	0.145*	–0.018	0.110	–0.06	0.079	0.573***	0.039
IC40	0.739***	–0.054	0.017	0.078	0.045	0.06	0.033	0.155	0.572***
IC41	0.640***	0.011	0.011	–0.022	0.011	0.000	0.000	–0.044	0.731***
IC42	0.536***	0.017	0.139*	–0.016	0.101	0.158*	0.031	–0.051	0.382***
IC43	0.631***	0.158*	–0.048	0.060	–0.042	0.025	0.054	0.063	0.549***

TW, teamwork; GS, goal setting; SS, social skills; PS, problem solving and decision making; ES, emotional skills; LS, leadership skills; TM, time management; IC, interpersonal communication. *p < 0.05, **p < 0.01, ***p < 0.001.

**TABLE 6:** Inter-factor correlations for the re-specified CFA and ESEM models.

	1	2	3	4	5	6	7	8
**Re-specified CFA Model**								
1. TW	−							
2. GS	0.433***	−						
3. SS	0.445***	0.435***	−					
4. PS	0.380***	0.608***	0.494***	−				
5. ES	0.370***	0.541***	0.556***	0.498***	−			
6. LS	0.424***	0.608***	0.488***	0.605***	0.508***	−		
7. TM	0.180***	0.544***	0.350***	0.540***	0.496***	0.490***	−	
8. IC	0.429***	0.466***	0.514***	0.528***	0.600***	0.565***	0.583***	−
**Re-specified ESEM Model**								
1. TW	−							
2. GS	0.284**	−						
3. SS	0.293**	0.265**	−					
4. PS	0.256*	0.510**	0.310**	−				
5. ES	0.183	0.321**	0.314**	0.289*	−			
6. LS	0.171*	0.402**	0.246**	0.385***	0.226*	−		
7. TM	0.070	0.429***	0.239**	0.406***	0.272**	0.309***	−	
8. IC	0.269*	0.264*	0.331***	0.367**	0.282*	0.245**	0.424***	−
α	0.71	0.79	0.71	0.74	0.71	0.70	0.83	0.72

TW, teamwork; GS, Goal setting; SS, social skills; PS, problem solving and decision making; ES, emotional skills; LS, leadership skills; TM, time management; IC, interpersonal communication. *p < 0.05, **p < 0.01, ***p < 0.001.

### Internal Reliability

Cronbach’s alphas for the current sample were calculated to assess the internal consistency of the LSSS subscales. Reliabilities for the initial subscales using the current data, ranged from 0.69 (Teamwork) to 0.83 (Time management), demonstrating acceptable internal consistency for all subscales (see Table 3; Kline, 2000). After re-specifying the model, the Cronbach’s alpha for Teamwork slightly increased to 0.71, whereas the alphas for Social Skills and Leadership decreased from 0.74 to 0.71 and from 0.80 to 0.70, respectively (see Table 6).

## Discussion

The LSSS was developed with the aim of creating a sport-specific measure of like skills development. While scores produced by the LSSS were found to be reliable and valid among samples of British youth sport participants (Cronin and Allen, 2017, 2018; Mossman and Cronin, 2019), only four known studies have examined the scale’s psychometric properties within other cultural contexts (AÇAK and DÜZ, 2018; Lim et al., 2019; Nascimento-Junior et al., 2020; Sabourin et al., 2020). With evidence of a growing interest in sport-based PYD programs across the African continent, research examining the reliability and validity of scores derived from existing measures of life skills, within this context, is highly needed. This would be the most reasonable step before determining if developing new or context-specific measures is necessary. As such, the main aim of this study was to examine the latent structure and reproducibility of scores on the LSSS in a sample of youth sport participants from Botswana and Ghana. Within the African context, current findings support an eight-factor re-specified model of the LSSS, consisting of 34 items and acceptable internal consistency.

Consistent with previous findings (AÇAK and DÜZ, 2018; Lim et al., 2019), some re-specification of the original LSSS model suggested by Cronin and Allen (2018) was needed for the scale to fit within the current cultural context. While the revised version of the scale consists of the original eight factors (Cronin and Allen, 2017; Teamwork, Goal setting, Social skills, Problem solving and decision making, Emotional skills, Leadership, Time management, and Interpersonal communication), only 34 items from the original scale were retained within this study. Given the multidimensionality of the LSSS, compared to CFA, ESEM provides methodological advantages for exploring the eight-factor structure of the LSSS (Marsh et al., 2014). Results from ESEM revealed a total of nine items that did not work as expected in the two African contexts, on subscales designed to measure Teamwork (items 2, 3, 6, and 7), Social skills (item 18), and Leadership (items 28, 29, 33, and 35). Following omission of these nine items, the re-specified model of the LSSS resulted in improved overall model-data fit compared to the original LSSS. It can therefore be concluded that while the aforementioned nine items of the LSSS do not generalize well within the present context, the results of this study provide support for the usefulness of the re-specified version of the LSSS in an African context, specifically in Botswana and Ghana.

Similar to previous cross-cultural examinations of the LSSS (AÇAK and DÜZ, 2018; Lim et al., 2019), the findings of this study can be interpreted in light of the present cultural context. Specifically, as it relates to Teamwork and Leadership, it is possible that the excluded items from these scales do not accurately reflect conceptualizations of Teamwork or Leadership within the Botswana and Ghana youth sport contexts. For example, this study found that three of the items designed to measure Teamwork (item 2: Help another team/group member perform a task; item 6: Suggest to team/group members how they can improve their performance: item 7: Change the way I perform for the benefit of the team/group) produced noticeable cross-loadings on the Leadership subscale. It is therefore possible that the items designed to measure Teamwork were interpreted as reflecting Leadership within the present context. Additionally, the items of the LSSS designed to measure Leadership may not align well with conceptualizations of leadership within the context of this study. Particularly, it appears that the conceptualization of Leadership reflects the collaborative behaviors that are characteristic of Teamwork. If this is true, then the previously reported shift in the conceptualization of leadership from being concerned with the personal possession of power, to an emphasis on social, collaborative, and relational attributes (Cullen-Lester and Yammarino, 2016; Ferkins et al., 2018) is likely the case within the present study. The primacy of these attributes in the conceptualization of youth leadership makes intuitive sense, especially in these contexts where the attributes are likely to be reinforced by the way youth sport programs are run and organized.

Measurement issues have been particularly relevant to PYD research, and more specifically within the context of sport (Sullivan et al., 2015). On one hand, it has been concluded that the lack of an operational definition for PYD has led to varied results for the influence of sport on youth development (MacDonald and McIsaac, 2016). On the other hand, measures of PYD tend to be multidimensional in nature, consisting of numerous factors and items [e.g., the Youth Experiences Survey for Sport (YES-S; MacDonald et al., 2012); Life Skills Transfer Survey (Weiss et al., 2014)]. This multidimensionality can lead to overlapping domains, as well as cross-loadings among items due to factors being conceptually related (Morin et al., 2016).

To provide an example, when the original 5-factor structure (Personal and Social skills, Initiative experiences, Cognitive skills, Goal setting, and Negative experiences) of the 37-item YES-S was examined among a sample of youth sport athletes, only 22 resulting items were retained that supported the scale’s factor structure (MacDonald et al., 2012). This led to the conclusion that only a minimal number of items were sufficient to assess PYD in sport and create a psychometrically sound instrument (MacDonald et al., 2012). The results of this study support this conclusion, as a more concise version of the LSSS resulted in improved fit. Considering that the LSSS is a relatively new measure that has not undergone extensive revisions and psychometric evaluation, there is a need for additional investigations into its psychometric properties cross-culturally, and across various sport contexts. It is possible that conceptualizations of the different life skills reported to develop through sport may vary based on the context-specific nature of sport (e.g., team vs. individual, recreational vs. competitive), the way youth sport programs are organized and run, and levels of commercial interests in youth sport.

Consistent with previous validations of the LSSS (Cronin and Allen, 2017; AÇAK and DÜZ, 2018; Lim et al., 2019; Nascimento-Junior et al., 2020; Sabourin et al., 2020), adequate internal consistency was observed for the eight subscales prior to, and following revision of the scale, though the alpha coefficients in this study were generally smaller than in previous studies. Within the present context, smaller alpha coefficients may indicate that while these items are adequate measures of the eight life skills, the items may benefit from modification or cultural adaptation to improve the scale’s internal consistency. When examining previous validation studies (Nascimento-Junior et al., 2020; Sabourin et al., 2020), following the adaptation of the LSSS for use within the specific cultural context, internal consistency reliabilities ranged from 0.74 to 0.92. As such, when establishing meaningful assessment tools, there appear to be methodological advantages to cross-cultural adaptations of existing instruments.

### Limitations and Suggestions for Future Research

Despite these contributions, this study is not without limitations. The small sample size should be noted as one of the limitations of this study, particularly given that the original LSSS model we tested consists of 43 items. This small sample prevented us from testing possible invariance in the meaning and structure of the scale by group membership. For example, this study recruited youth sport participants from a wide age range (12–21 years), which may have resulted in a heterogenous sample with regard to levels of cognitive, social, emotional, and other domains of development. As such, differing levels of maturation may have influenced the perception of the development of the eight life skills measured by the LSSS. While insufficient power due to a small sample limited the ability to assess measurement invariance in the current study, future research may account for these differences by examining measurement invariance of the LSSS across varying age groups.

Undoubtedly, the validation of psychological measures is an ongoing process (Nunnally and Bernstein, 1994). While this study established factorial validity of the LSSS, other sources of validity evidence, such as construct and predictive validity were not assessed, and should be considered for examination in future research within this context. Specifically, to provide further evidence for the applied utility of the LSSS, future studies should aim to examine the extent to which the scale converges with other similar measures of sport-specific life skills (e.g., the YES-S; MacDonald et al., 2012) and PYD (e.g., the PYD Short Form Scale; Geldhof et al., 2014). The extent to which scores on the LSSS predict success on measures of academic achievement, well-being, and various sport-related psychosocial variables (e.g., performance, motivation) is also a worthy avenue for future exploration. Importantly, future research aiming to examine the psychometric properties of the LSSS within the African context may also seek to provide evidence for temporal stability, and confirm the factor structure, as found in the present study, among additional samples of African youth sport participants. Additional investigations into the conceptualization of sport leadership and teamwork, within the African youth sport context, may also provide fruitful insights for later modifications or adaptations of the LSSS for use within this, as well as other populations.

## Conclusion

Within the broader context, this study adds evidence for the suitability of the LSSS in evaluating the eight life skills reported to develop through sport (Cronin and Allen, 2017). Specifically, the current study indicates that a sample of youth sport participants from Ghana and Botswana perceived the development of social, emotional, teamwork, goal setting, leadership, as well as problem solving and decision making, time management, and interpersonal communication skills, through their sport participation. Given these findings, the use of this measure within the African youth sport context may provide additional insights into the role of sport as an avenue for adaptive development, as is emphasized by the PYD and positive psychology frameworks. More so, considering the crucial role of social and emotional skills for later success across academic, life, and career domains (Schonert-Reichl, 2017), the LSSS may serve as a valuable tool for examining youth sport as a vehicle for the development of these highly beneficial skills.

Importantly, this study provides validity evidence for the scores produced by the LSSS within two African countries that are culturally different and geographically distant from one another. This instrument can therefore aid researchers and sport practitioners in the monitoring and evaluation of youth sport programs and their effectiveness in fostering PYD.

## Data Availability Statement

The data presented in this study can be found in online repositories. The link to the repository and accession number is https://doi.org/10.6084/m9.figshare.17049860.

## Ethics Statement

The studies involving human participants were reviewed and approved by the Institutional Review Boards of the University of Botswana and the University of Ghana. Written informed consent to participate in this study was provided by the participants’ legal guardian/next of kin.

## Author Contributions

LM led the conception, design, writing, and revisions to the manuscript. CR contributed to the writing and revisions of the manuscript. SK conducted the statistical analyses and wrote the results section. TT, TM, CA, and RO contributed to the conception, design, data collection, curation, and revisions. All authors contributed to the article and approved the submitted version.

## Conflict of Interest

The authors declare that the research was conducted in the absence of any commercial or financial relationships that could be construed as a potential conflict of interest.

## Publisher’s Note

All claims expressed in this article are solely those of the authors and do not necessarily represent those of their affiliated organizations, or those of the publisher, the editors and the reviewers. Any product that may be evaluated in this article, or claim that may be made by its manufacturer, is not guaranteed or endorsed by the publisher.
